# UBE2L3, a Partner of MuRF1/TRIM63, Is Involved in the Degradation of Myofibrillar Actin and Myosin

**DOI:** 10.3390/cells10081974

**Published:** 2021-08-03

**Authors:** Dulce Peris-Moreno, Mélodie Malige, Agnès Claustre, Andrea Armani, Cécile Coudy-Gandilhon, Christiane Deval, Daniel Béchet, Pierre Fafournoux, Marco Sandri, Lydie Combaret, Daniel Taillandier, Cécile Polge

**Affiliations:** 1 Université Clermont Auvergne, INRAE, UNH Unité de Nutrition Humaine, F-63000 Clermont-Ferrand, France; dulce.peris-moreno@inrae.fr (D.P.-M.); melodie.malige@inrae.fr (M.M.); agnes.claustre@inrae.fr (A.C.); cecile.coudy-gandilhon@inrae.fr (C.C.-G.); christiane.deval@inrae.fr (C.D.); daniel.bechet@inrae.fr (D.B.); pierre.fafournoux@inrae.fr (P.F.); lydie.combaret@inrae.fr (L.C.); daniel.taillandier@inrae.fr (D.T.); 2 Department of Biomedical Sciences, Venetian Institute of Molecular Medicine, University of Padua, 35100 Padova, Italy; andre.arma88@gmail.com (A.A.); marco.sandri@unipd.it (M.S.)

**Keywords:** MuRF1/TRIM63, skeletal muscle atrophy, glucocorticoids, UBE2L3/UbcH7, alpha-actin, myosin, ubiquitinating enzymes, contractile proteins, MicroScale-Thermophoresis

## Abstract

The ubiquitin proteasome system (UPS) is the main player of skeletal muscle wasting, a common characteristic of many diseases (cancer, etc.) that negatively impacts treatment and life prognosis. Within the UPS, the E3 ligase MuRF1/TRIM63 targets for degradation several myofibrillar proteins, including the main contractile proteins alpha-actin and myosin heavy chain (MHC). We previously identified five E2 ubiquitin-conjugating enzymes interacting with MuRF1, including UBE2L3/UbcH7, that exhibited a high affinity for MuRF1 (K_D_ = 50 nM). Here, we report a main effect of UBE2L3 on alpha-actin and MHC degradation in catabolic C2C12 myotubes. Consistently UBE2L3 knockdown in Tibialis anterior induced hypertrophy in dexamethasone (Dex)-treated mice, whereas overexpression worsened the muscle atrophy of Dex-treated mice. Using combined interactomic approaches, we also characterized the interactions between MuRF1 and its substrates alpha-actin and MHC and found that MuRF1 preferentially binds to filamentous F-actin (K_D_ = 46.7 nM) over monomeric G-actin (K_D_ = 450 nM). By contrast with actin that did not alter MuRF1–UBE2L3 affinity, binding of MHC to MuRF1 (K_D_ = 8 nM) impeded UBE2L3 binding, suggesting that differential interactions prevail with MuRF1 depending on both the substrate and the E2. Our data suggest that UBE2L3 regulates contractile proteins levels and skeletal muscle atrophy.

## 1. Introduction

Many diseases (cancer, sepsis, heart failure, kidney diseases, etc.) are associated with cachexia, a catabolic state that is characterized by a dramatic skeletal muscle wasting. This sustained wasting consequently leads to muscle weakness and frailty of patients, impairs movement, decreases autonomy and also has detrimental metabolic consequences for muscle and other organs. Hence, almost one-third of cancer death is the consequence of cachexia [1,2] highlighting the dramatic deleterious aspect of muscle atrophy. It is now admitted that an increased skeletal muscle proteolysis is the main determinant of muscle wasting, and, among the proteolytic systems, the ubiquitin proteasome system (UPS) is believed to be the main actor of contractile protein degradation [3,4,5,6,7,8,9,10].

Proteins degraded by the UPS are first tagged with a chain of ubiquitin (Ub), an 8.5 kDa protein, with each Ub moiety being linked together mainly via their lysines 11 or 48 [11]. This labeling results from an E1–E2–E3 enzymatic cascade, where one of the two E1 ubiquitin-activating enzymes activates and transfers Ub to the active site cysteine of one the 40 E2 Ub-conjugating enzymes. The E2 enzyme interacts thereafter with an E3 Ub ligase (more than 700 have been predicted in the human genome), whose role is to recognize the substrate to be labeled. E2 and E3 enzymes, through their interaction, confer the targeting specificity of the UPS, thanks to the ability of the E2s to interact with several E3s and vice versa [12].

Among the E3 ligases involved in muscle mass homeostasis [13], muscle-specific RING finger protein 1 (MuRF1), also called TRIM63, is particularly important. This muscle-specific RING-type E3 ligase is overexpressed in more than 25 catabolic conditions [14]. As such, MuRF1 is a founding member of the atrogene family and is a reliable marker of skeletal muscle atrophy [14,15]. Mice with null alleles for the MuRF1 gene are partly resistant to skeletal muscle atrophy upon numerous catabolic conditions [8,16,17,18]. The contractile proteins represent up to 70% of the muscles’ cell protein content [19], and MuRF1 is the only E3 ligase known to target several ones (a-actin, myosins, troponins and telethonin) for degradation during catabolic conditions [7,8,9,10,20,21].

MuRF1, as a RING-type E3, has no activity per se and is thus dependent on an E2 enzyme that brings the catalytic activity [22]. Using complementary biochemical and in cellulo approaches, we recently identified five E2 enzymes expressed in muscle that interacted with MuRF1, namely UBE2E1, UBE2G1, UBE2J1, UBE2J2 and UBE2L3 [23]. Moreover, we have shown that the presence of a substrate (telethonin) allosterically stabilized MuRF1–E2E1 and MuRF1–E2J1 interactions [23], suggesting that these MuRF1–E2 pairs are responsible for telethonin degradation.

Among the MuRF1-interacting E2s, UBE2L3/UbcH7 (referred to here as E2L3 throughout the text) particularly attracted our attention as it presented the highest affinity for MuRF1 [23]. Like all but one E2 enzymes, E2L3 is not an atrogene; that is, its mRNA expression is not up- or downregulated in muscle during catabolic states [23]. However, E2L3 is highly abundant in skeletal muscle (human proteome Atlas, https://www.proteinatlas.org (accessed on 28 July 2021); [24]) E2L3 was initially thought to work only with HECT- and RBR-type E3 ligases [24], but other studies have revealed that E2L3 can also cooperate with CHIP (a U-box E3 ligase) [25] and with RING-type E3 ligases like c-Cbl [26,27,28], RNF213 [29] and MYCBP2/PHR1 [30]. Recently, an Activity-Based Probe (ABP) against E3 ligases has been developed, based on E2L3 acting as a “suicide” substrate covalently trapping E3 ligases [30,31] in neuroblastoma SH-SY5Y cell extract. The authors identified 40 HECT/RBR E3 ligases (80% of this category) but also 33 RING E3 ligases [30], thus underscoring the versatility of function of E2L3.

E2L3 has been linked to different pathological conditions. Polymorphisms in the genomic locus of E2L3 have been associated with multiple autoimmune diseases, such as lupus erythematosus [32,33,34], Crohn’s disease [35] or psoriasis [36] (for a review, see Reference [22]). E2L3 alterations could involve the cooperation between the E3 ligase complex LUBAC and E2L3 leading to the hyperactivation of the NF-κB pathway [22]. E2L3 is also implicated in cell-cycle control by regulating the entrance and progression through the different phases (G1, S, G2 and M) [37,38]. Besides, E2L3 stabilizes p27Kip1, an inhibitor of cyclin dependent kinase [39]. Such control of the cell cycle has linked E2L3 to cancer as described for cervical cancer [40] or as reported by gene correlation analysis in rectus abdominis muscle from cancer patients [41]. Moreover, via Ub-dependent degradation, E2L3 also controls 53BP1 protein levels, a main actor of DNA double-strand breaks repair [42,43], adding further evidence of an implication of E2L3 in cancer. E2L3 is also one of the E2 enzymes shown to collaborate with the E3 ligase PARKIN, a regulator of mitophagy, involved in Parkinson disease and potentially a tumor suppressor [44].

However, E2L3 functions in skeletal muscle and its putative involvement during muscle atrophy is not known. In this study, we showed that E2L3 is involved in the degradation of the main contractile proteins, alpha-actin (a-actin) and myosin heavy chain IIa (MHCIIa) in catabolic C2C12 myotubes. We also found that in vivo E2L3 may be involved in muscle atrophy processes in vivo. We used complementary interactomics approaches to characterize the interaction between MuRF1 and E2L3 in the presence of MuRF1 substrates, i.e., a-actin or myosin. We found that myosin inhibited MuRF1–E2L3 interaction. This approach also allowed us to demonstrate that MuRF1 has a better affinity for filamentous than monomeric a-actin.

## 2. Materials and Methods

### 2.1. Constructs

Using Superscript II and Platinum Pfx DNA polymerase (Invitrogen), we amplified by RT-PCR rat MuRF1, murine alpha-actin (a-actin), myosin heavy chain IIa (MHCIIa; MYH2 gene), the DNA fragment encoding amino acids 2–255 WT MHCIIa, UBE2E1 and UBE2L3 from either rat soleus muscles or murine C2C12 skeletal muscle cells. Then cDNAs encoding for E2 proteins and MuRF1 were cloned in the expression vectors pET28a (Novagen) and pGEX6P3, respectively, for the production of recombinant proteins in *Escherichia coli* (*E. coli*, BL21(DE3)). For mammalian in cellulo assays, UBE2L3 was sub-cloned in the expression vector pcDNA3.1 (Thermofisher), as described for flag-a-actin, MHCIIa-Flag, Myc-MuRF1 [45]. For yeast three-hybrid, UBE2 enzymes, MuRF3 and Large-T cDNAs were cloned in pGADT7 vector (containing the activation domain of GAL4) (Clontech). The pBridge vector (Clontech) allows the expression of two proteins: MuRF1 was cloned in the first Multiple Cloning Site (MCS I) in fusion with the binding domain of GAL4, and MHCIIa (residues 1–255) was cloned in the MCSII, leading to the pBridge::MuRF1/MHCIIa plasmid.

### 2.2. Antibodies and Proteins

The antibodies used for Western blot were monoclonal Anti-Proliferating Cell Nuclear Antigen (PCNA) (clone PC10 Sigma-Aldrich P8825; 1:3000), monoclonal anti-alpha-actin (Sigma-Aldrich HHF35; 1:5000), monoclonal anti-myosin heavy chain type IIa (DSHB SC-71; 1:1000), monoclonal anti-Flag (Sigma-Aldrich F1804; 1:6000) and polyclonal anti-UBE2L3 (Sigma; 1:1000). The secondary antibodies used were anti-mouse (IRDye^®^ 800 CW, Licor 92632210 or IRDye^®^ 680, Licor 92632220;), anti-goat (IRDye^®^ 800 CW, Licor 92632214) and anti-rabbit (IRDye^®^ 800 CW, Licor 92632221). The antibody used for skeletal muscle cross-sectional analysis was anti-laminin-α1 (L9393) from Sigma.

Heavy Meromyosin (HMM; Cat. # MH01) (including the first 800 amino acids of MHC plus the two pairs of light chains) and alpha-actin (Catalogue No. AKL99) purified from rabbit skeletal muscle were purchased from Cytoskeleton, Inc., Denver, CO, USA. Alpha-actin was reconstituted in General Actin Buffer (5 mM Tris-HCl pH 8.0, 0.2 mM CaCl2 and 0.2 mM ATP) supplemented with 0.2 mM ATP and 0.5 mM DTT, following the supplier’s instructions. Then 1/10th volume of Polymerization Buffer (500 mM KCl, 20 mM MgCl2, 10 mM ATP) was added to induce the polymerization of a-actin. UBE2G2, UBE2N, UBE2V2, UBE2Z and UBE2K were purchased from LifeSensors. UBE2D2 and UBE2C were purchased from Enzo Life Sciences.

### 2.3. Protein Expression and Purification

GST-MuRF1 was expressed and purified by using sepharose 4B affinity matrix (GE Healthcare), as described in Reference [9]. UBE2A, UBE2B, UBE2E1, UBE2G1 and UBE2L3 were produced in BL21(DE3) *E. coli*, as his-tag fusion proteins and purified on Ni-NTA agarose matrix (Qiagen), as described in Reference [23].

### 2.4. Cell Culture and Knockdown Experiments

The C2C12 cell line was provided by LGC promochem, a European partner of ATCC. Cells were grown at 37 °C, under 5% CO_2_. Murine myocytes (C2C12) were grown in proliferation medium (PM): Dulbecco’s Modified Eagle Medium (DMEM) with 4.5 g/L glucose (ThermoFisher, D6546) supplemented with 10% FBS (ThermoFisher 10270-106), 2% L-Glutamine (Sigma-Aldrich G7513), 1% 14600X Non-Essential Amino Acids (NEAA) (ThermoFisher, 11140-035), 1% Penicillin–Streptomycin (ThermoFisher, 15140-122) and 1% Plasmocin (InvivoGen, ant-mpp). Once cells were 70–80% confluent, they were washed with 1X PBS and shifted to differentiation medium (DM) containing 2% horse serum instead of FBS (H1270, Sigma).

Knockdown (KD) experiments were performed in C2C12 myotubes grown in 24-well plates. C2C12 myotubes were transfected or not (mock transfection, negative control) at day 5 of differentiation (d5), with shRNAs (MISSION^®^ shRNA, Sigma) targeting either MuRF1 (sh-MuRF1-1, TRCN0000254632; sh-MuRF1-2, TRCN0000254634), UBE2L3 (sh-UBE2L3-1, TRCN0000040824; sh-UBE2L3-2 TRCN0000040825) or no known protein sequence (scrambled-shRNA, negative control, SHC002MISSION), using a NEPA21 electroporator (SONIDEL Limited, Ireland). To perform electroporation, pLKO.1 constructs were diluted at 0.46 µg/µL in OptiMEM (ThermoFisher Scientific, Waltham, MA, USA) when singly transfected and at 0.23 µg/µL for doubly transfected cells. Co-transfection was performed with two different pLKO.1 constructs targeting the same gene of interest to ensure efficient KD (>60%). Mock transfection was conducted by only using OptiMEM. A 24-well plate was used for each condition; the cells from four wells were combined for obtaining enough amount of proteins, which ended up with a maximum of six independent samples. The experiment has been repeated twice.

Briefly, C2C12 myotubes were rinsed twice with 500 µL of 1× PBS. Then, 300 µL of OptiMEM or OptiMEM+plasmids was added. Electroporation was performed by using an adherent cell electrode (SONIDEL Limited, Ireland), following the instructions of the manufacturer with the following parameters: 2 poring pulses, 225 V (5 ms, 10% decay, 50 msec interval); 5 transfer pulses, 30 V (50 ms, 40% decay, 50 ms interval). Thereafter, the DNA solution was removed, and 500 μL of fresh DM deprived of antibiotic and serum was added to the cells. Cells were incubated for 90 min at 37 °C, under 5% CO_2_, and the medium was then replaced by DM containing or not dexamethasone (Dex) (1 μM). After 48 h of Dex treatment, cells were washed in cold 1X PBS twice, and 300 µL of lysis buffer 1× PBS, 5 mM EDTA, 1 mM phenylmethylsulfonyl fluoride, 10 mM NEM, 1% Triton X-100/anti-proteases (Protease Inhibitor Mixture/Sigma), 1 u/mL DNAseI and 10 mM N-Ethylmaleimide (NEM) were added. Then the cells were scraped off the plate on ice and homogenized by sonication for 2 × 30 s, at maximum power, using a UP50H sonicator (Hielscher, Teltow, Germany), as previously described [45]. Cell lysates were then centrifuged at 10,000× *g*, for 10 min, at 4 °C, and the supernatant (soluble fraction) was kept at −80 °C until use. The pellets enriched in myofibrillar proteins were resuspended in a homogenization buffer and sonicated to solubilize the proteins. The protein concentration was measured by absorption spectrophotometry (OD 562 nm), using the BCA kit (Pierce, Rockford, IL, USA) with BSA as a standard. For Western blots, fluorescence of the secondary antibodies was detected by using a LI-COR Odyssey Imager (LI-COR Biosciences, Lincoln, NE, USA), and blots were quantified by using the LI-COR Odyssey software.

### 2.5. Animals Overexpression and Knockdown Experiments

In vivo transfection was performed as previously described [45]. The experiments were conducted in accordance with the French National Research Council Guide for the Care and Use of Laboratory Animals. National authorization to perform animal experiments for this project was obtained (authorization #9204 for project 2017042115497506). All animals were maintained in a temperature-controlled room (22 ± 1 °C) with a 12:12 h light:dark cycle. Three-month-old male C57BL/6 mice were housed for 1 week in standard environment and then submitted or not to Dex treatment at 5 mg/kg/day for five days. In vivo knockdown or overexpression was performed as previously described by electroporation [46]. For overexpression, the coding sequence of mouse E2L3 was sub-cloned into pcDNA3.1 mammalian expression vector. For knockdown experiments, a target finder and design tool (Invitrogen) was used to identify target regions in the mouse E2L3 genes amenable to shRNA. The sequences of shRNAs targeting E2L3 were cloned into the BLOCK-iT Pol II miR RNAi Expression Vector Kit with EmGFP (Invitrogen number K4936-00). The sequences used are shown in Supplementary Materials Table S1 and a mixture of two shRNAs was used for in vivo transfection. As a negative control, the pcDNA6.2-GW/EmGFP-miR-neg control was used according to manufacturer’s instructions. Mice were transfected four days prior to Dex treatment with either a scramble shRNA, a mixture of two shRNA directed against E2L3 or a plasmid encoding for E2L3. At the end of the experiment, animals were euthanized by cervical dislocation. The skeletal muscles (Tibialis anterior) were excised, frozen in liquid nitrogen-cooled isopentane and stored at −80 °C until use. Cross-sections were labeled with anti-laminin-α1 for determining fiber cross-sectional area (CSA), using the ImageJ v. 1.53J software. Four different zones and a minimum of 380 fibers (380–1200) were used for each muscle. The plasmids encoding for the shRNAs also expressed emGFP, which allowed us to identify the transfected cells and estimate the efficiency of transfection (≈50%; see Supplementary Materials Figure S2); n = 6 mice per group.

### 2.6. Binding Constant Determination by Fluorescence Quenching

Fluorescence quenching experiments were performed by using a NanoTemper ^®^ Monolith NT.115 (NanoTemper Technologies, Germany) with blue/red filters. Red fluorescent dye NT-647 was covalently bound to GST-MuRF1 by amine coupling (excitation 650 nm; emission 670 nm), using the Protein Labeling Kit REDNHS (NanoTemper Technologies). Labeled MuRF1 concentration was kept constant at 5 nM, while the concentration of the ligand varied. A 16-sample serial dilution of the ligands was made from 500 nM to 15.3 pM for HMM, from 6.25 μM to 0.763 nM for filamentous a-actin, from 25 μM to 0.763 nM for globular a-actin, from 20 µM to 38.1 pM for E2L3 and from 9 μM to 0.275 nM for E2E1. Buffer solutions were tested to determine the best buffer composition based on protein stability and homogeneity within capillaries. Finally, experiments were realized in 50 mM HEPES buffer pH 7.4, 150 mM NaCl, 0.005% v/v Tween20, 0.1% dextran. Prepared samples were incubated in the dark, at 22 °C, during 30 min, to let the interaction stabilize; they were then loaded into premium treated capillaries for measurements, using 40% MST power at 25 °C. The Monolith allowed us to measure interactions either by microscale thermophoresis (MST) or by the change of fluorescence of the fluorescent partner. Indeed, in some cases, ligand binding can change the photobleaching properties of the fluorescent target. In those cases, the photobleaching signal has to be used for calculation of affinity. Both MST and photobleaching depend on the ligand concentration. In the case of MuRF1, data from fluorescence quenching were evaluated instead of MST traces. An affinity measurement was considered reliable when the signal-to-noise ratio was ≥10. All experiments were repeated at least three times for each measurement. Graphs present the averages obtained with the different replicates. Data analyses were performed by using the MO.Affinity Analysis software (version 2.3, NanoTemper Technologies).

### 2.7. Yeast Three-Hybrid Experiments

We used the “Matchmaker™ Gold Yeast Two-Hybrid System” (Y2H) from Clontech, as described in Reference [23]. The pBridge vector was used to perform yeast three-hybrid (Y3H) experiments, in combination with the AD fusion vector pGADT7. Growth on selective plates (medium lacking leucine, tryptophan and histidine and supplemented with 20 mM Aureobasidin A and 2.5 mM 3-Amino-1,2,4-triazole (-LTH + Aureo + 3-AT)) was followed over a period of 21 days. The drug-reporter-gene AUR1-C expression confers strong resistance to the highly toxic drug Aureobasidin A and exhibits very little background activity. The number of clones analyzed were 12 for MuRF1/E2D2, 10 for MuRF1-MHCII/E2D2, 16 for MuRF1/E2L3 and 10 for MuRF1-MHCII/E2L3.

### 2.8. Statistical Analysis

Results are expressed as means ±SEM. Comparisons between groups were performed first by using a one-way ANOVA (analysis of variance). When statistical significance was reached, it was followed by multiple post hoc Tukey’s tests. Analyses were performed by using GraphPad Prism 9.0.1 software.

## 3. Results

### 3.1. E2L3 Knockdown Preserves a-Actin and MHCII in Myofibrillar Fractions in Catabolic C2C12 Myotubes

**Figure 1:**
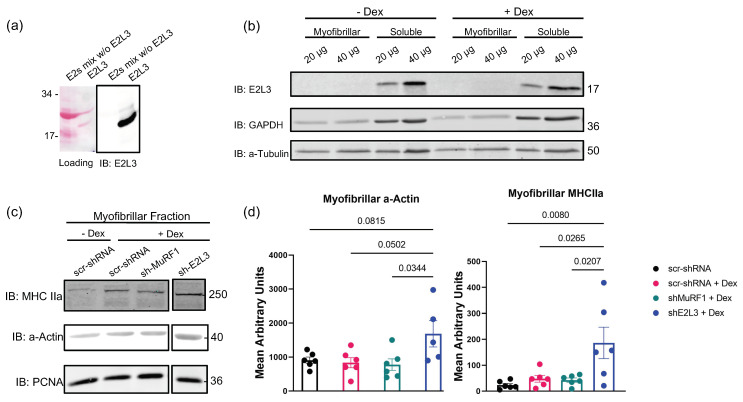
E2L3 knockdown preserved a-actin and MHCII in myofibrillar fraction in catabolic C2C12 myotubes. (**a**) A mixture of 11 recombinant E2 enzymes (E2 mix w/o E2L3) was used to address the selectivity of E2L3 antibody. A total of 1 μg of each E2 enzyme was used. Only E2L3 was recognized by the antibody used. (**b**) E2L3 is mainly present in the soluble fraction of C2C12 myotubes. C2C12-soluble and myofibrillar-enriched fraction were assayed for E2L3, GAPDH and alpha-tubulin (a-tubulin) by immunoblotting (IB) (20 and 40 μg of total proteins were loaded). GAPDH was used as marker of soluble-enriched fraction and a-tubulin as loading control. (**c**) Myofibrillar proteins were assayed for a-actin and MHCIIa levels by immunoblotting (15 μg of total proteins were loaded). C2C12 myotubes were cultured in 24-well plates, treated with Dex 1 μM and electroporated, as explained in Section 2, with two shRNA directed against E2L3 or MuRF1. (**d**) Densitometry analysis was performed on data from (**c**). Values were expressed after normalization with the loading control PCNA. Values are means SEM for n = 4 to 6 per group; *p*-values are indicated within the graph.

For simplification in this report, UBE2 proteins are called E2; for example, UBE2L3 is referred to as E2L3. As a strong E2 interactor of MuRF1, E2L3 appeared to be a promising candidate to participate with MuRF1 in the targeting of contractile proteins. Until now, only a single study has reported the E2L3 protein to be expressed in gastrocnemius and Tibialis anterior muscles [47]. E2L3 belongs to the class I E2 enzymes, which means it contains only the catalytic domain (UBC fold) that characterizes E2 enzymes, without N- or C-terminal extension [23]. Due to the presence of a common UBC fold in all E2s, we thus first checked the selectivity of the E2L3 antibody (Figure 1a). We then showed that E2L3 was expressed in C2C12 myotubes at the protein levels (Figure 1b). E2L3 was mainly found in the soluble fraction, as expected for a protein that is not tightly bound to the sarcomere, which indicates that sonication was able to break the labile interactions prevailing between E2 and E3 enzymes (Figure 1b). We then studied its impact on C2C12 myotubes that were treated with dexamethasone (Dex, 1 μM) to induce a catabolic situation, as demonstrated by the increased expression of MuRF1 mRNA (see Supplementary Materials Figure S1a). As already observed in this model [45], MuRF1 knockdown (KD) did not modify myofibrillar a-actin and MHCIIa levels, two of its substrates (Figure 1c,d), compared to controls (scr-shRNA ± Dex). As MuRF1 targets several myofibrillar proteins (MHC, a-actin, troponin, etc.), the myofibrillar proteins loaded on SDS-PAGE are highly proportional to the levels of MuRF1 targets. Moreover, a single study observed a strong decrease of both MHCI and MHCII by using 100 µM Dex, a suprapharmacological dose that generally is not used in C2C12 cells (0.1–1 µM is the common range) [8]. In our study, we used a moderate catabolic state close to what can happen in vivo, which may also explain why we were not able to detect MHC and a-actin levels modification in C2C12 myotubes. It is noteworthy that Dex plasma concentration of patients receiving a clinical dose of Dex (8 mg) is below 0.2 µM [48].

We then addressed the impact of E2L3 KD (shE2L3) on individual sarcomeric proteins in Dex-treated C2C12 myotubes; scramble shRNA and MuRF1 KD (shMuRF1) were used as controls. The KD of E2L3 significantly decreased E2L3 levels (Supplementary Materials Figure S1) and stabilized a-actin (+200%, *p* < 0.05) and MHCIIa (+380%, *p* < 0.03) in the myofibrillar-enriched fraction (Figure 1c,d), indicating that E2L3 is involved in the degradation of myofibrillar a-actin and myosin during Dex treatment. As the impact of E2L3 on a-actin and myosin is greater than the one of MuRF1, at least part of E2L3’s role may be independent of MuRF1.

### 3.2. E2L3 Overexpression Aggravates Muscle Atrophy and E2L3 KD Induces Hypertrophy in Dex-Treated Mice

**Figure 2:**
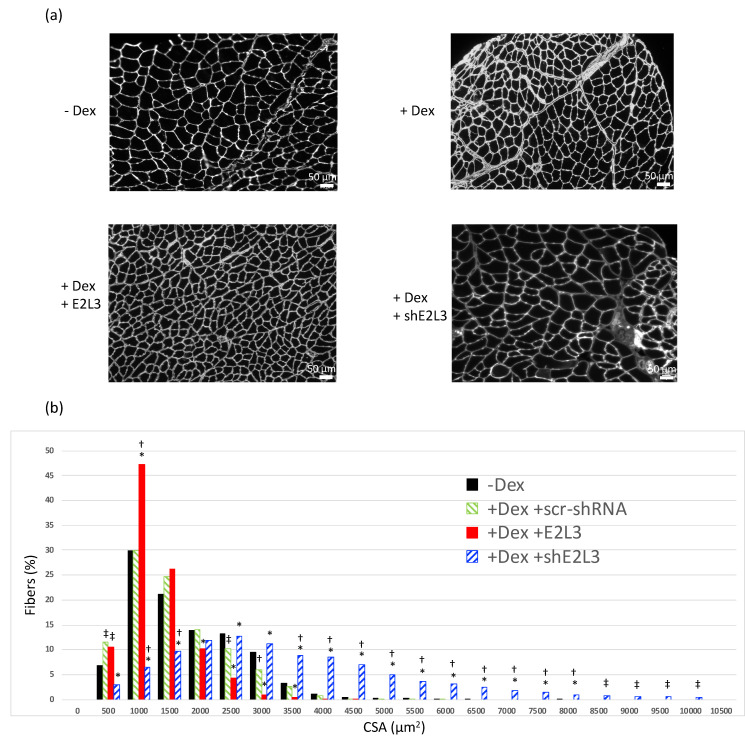
E2L3 KD and overexpression modify myofiber cross-sectional area (CSA). Mice treated or not with Dexamethasone (Dex 5 mg/kg/day) were transfected with either a scramble shRNA (scr-shRNA) targeting no known coding sequence, a mixture of two shRNAs (siE2L3, knockdown) directed against E2L3 or a plasmid encoding for E2L3 (E2L3, overexpression). Sh-encoding plasmids also encoded for emGFP, which allowed us to estimate the efficiency of transfection (typically around 50%; see Supplementary Materials Figure S2). Tibialis anterior muscle cross-sections were labeled with anti-laminin-α1 for determining CSA (see Section 2 for details). Four different zones and a minimum of 380 fibers were used for each muscle. (**a**) Representative cross-sections of T. anterior from each group are shown. Scale bar, 50 µm. (**b**) CSA analysis from n = 6 mice and a total of 17,945 fibers were analyzed, using a one-way ANOVA. Fibers distribution was analyzed within each muscle by grouping CSAs by 500 µm^2^ steps. A global shift towards lower CSA was observed when E2L3 was overexpressed in muscles. By contrast, larger CSA were observed in E2L3 KD muscles when compared to controls; n = 6 mice per group. * Statistically different from scr-shRNA group, *p* < 0.05. Statistically different from non-treated (-Dex) group, ^†^ *p* < 0.05, ^‡^ *p* < 0.1).

The results obtained on C2C12 myotubes prompted us to study the effect of E2L3 KD and overexpression in vivo. Dex-treatment induces skeletal muscle atrophy in vivo as witnessed by a decrease in cross sectional area (CSA), mainly in fast twitch muscles [45]. In our conditions, mice treated for five days with Dex (5 mg/kg/day) exhibited a moderate but significant switch of fibers towards smaller CSA in the T. anterior muscle (Figure 2). Interestingly, overexpression of E2L3 aggravated fiber CSA decrease, indicating an increased atrophy upon Dex treatment.

For further investigating the role of E2L3, we transfected T. anterior muscle with plasmids encoding for shRNAs directed against E2L3 Table S1 that also expressed emGFP. This allowed estimating the average efficiency of cell transfection (around 50%) in scr-shRNA and siE2L3 transfected muscles (Supplementary Materials Figure S2). By contrast with overexpression, the KD of E2L3 completely reversed muscle atrophy and even increased the percentage of large fibers when compared to muscles not subjected to Dex-treatment (Figure 2). Altogether, these data suggested an important role of E2L3 in muscle cell physiology.

### 3.3. E2L3 Interaction with MuRF1 Complexed to Filamentous Actin or Myosin

Since E2L3 was implicated in the degradation of the main contractile proteins in C2C12 myotubes and in the T. anterior muscle cell size, we then wondered whether this action could be linked to MuRF1. According to our previous report showing that telethonin (a MuRF1 substrate) allosterically stabilized E2E1–MuRF1 and E2J1–MuRF1 duos [23], we addressed whether E2L3–MuRF1 interaction could also be strengthened by the presence of a-actin and/or MHC, two substrates of MuRF1 [7,8,9].

#### 3.3.1. An Innovative Approach to Measure Proteins Interaction in Solution

**Figure 3:**
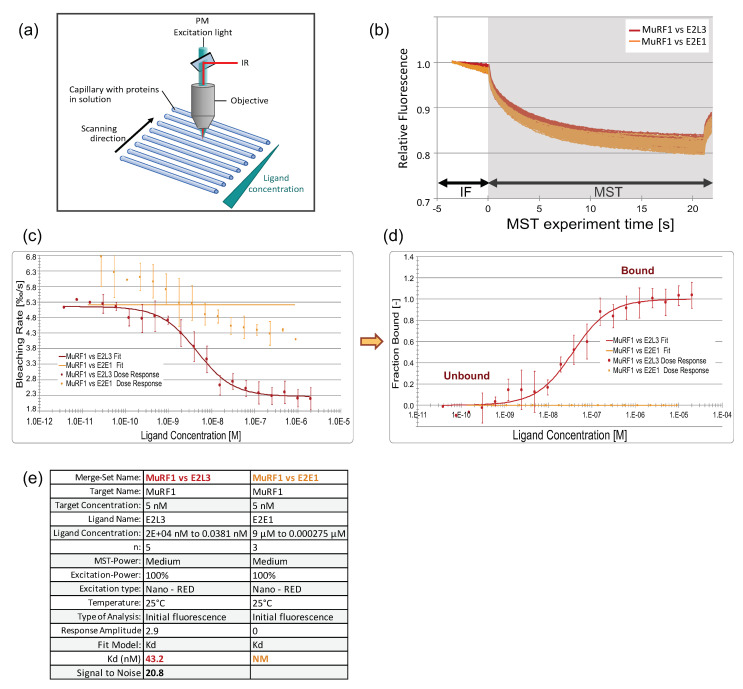
MuRF1–E2L3 affinity determination, using photobleaching rate. (**a**) Principle of the Monolith (adapted from Reference [50]). A fluorescent protein in solution in capillaries is excited with a UV LED; the fluorescence is recorded by a photomultiplier (PM) and plotted against time to get an MST (MicroScale-Thermophoresis) trace chart. (**b**) The fluorescent NT647–MuRF1 was mixed with the putative partner subjected to a 16-sample serial dilution. Variation of MuRF1 fluorescence was followed over time, in the presence of E2s; E2L3 (red), an interacting E2, and E2E1 (orange), a non-interacting E2 in this condition. When ligand binding changes the photobleaching properties of the fluorescent target, which is visible by the decrease in the initial fluorescence (IF) zone of the graph (t = −5 to 0 sec, before the Infra-Red (IR) laser was switched on), photobleaching rate has to be used for calculation of the affinity, instead of MST. (**c**) Dose–response curves are expressed in bleaching rate and plotted against E2s concentrations. (**d**) Transformation of dose–response curves from (**c**), in fraction bound for easier comparison between curves. (**e**) Dataset overview; n, number of replicates; NM, not measurable. A signal-to-noise >10 indicates a reliable result.

We used a highly sensitive interactomic approach that allowed us to study interactions between more than two proteins potentially forming a complex in solution, and more, not constrained by being bound to a support. We employed the NanoTemper ^®^ Monolith NT.115, allowing affinity determination from either photobleaching rate or MicroScale-Thermophoresis (MST) data (Figure 3a,b) [49,50].

Using the Monolith NT.115, we first set up the measurement conditions by using this approach by verifying the E2L3–MuRF1 and E2E1–MuRF1 interactions (Figure 3 and Supplementary Materials Figure S3) that we previously characterized by using Surface Plasmon Resonance (SPR) [23]. GST–MuRF1 was covalently labeled with a red fluorescent dye and first mixed with a 16-sample serial dilution of the ligand E2L3 (from 2 μM to 0.038 nM). The binding of E2L3 on MuRF1 led to the quenching of the initial fluorescence (IF white zone in Figure 3b) before switching on the infrared laser, rendering mandatory the use of fluorescence quenching data for the calculation of affinity. The change in bleaching rate was plotted against E2L3 concentration (Figure 3c) and this dose–response curve was fitted to derive the binding affinity constant (K_D_). The K_D_ was calculated by using the saturation binding curve at equilibrium. Alternatively, this curve can be transformed in fraction bound for easier comparison between assays (Figure 3d); state “0” corresponds to free MuRF1, while state “1” represents MuRF1 fully complexed with the ligand. This type of graph is used throughout the manuscript to present and compare the results.

Using this approach, we found an affinity of 43.2 nM for the MuRF1–E2L3 interaction, with a signal-to-noise >10 validating the reliability of the data (Figure 3e). This was consistent with the affinity that we previously estimated at 50 nM, using SPR [23]. We performed a similar experiment by using E2E1 as a ligand (Figure 3b), and the determination of K_D_ was not possible (Figure 3c–e), as it was the case using SPR [23]. Indeed, we previously showed that the MuRF1–E2E1 complex is not stable and needs the presence of a substrate to be stabilized [23]. This instability can be observed in Figure 3c, where some interaction can be detected but with high variability, thus preventing the fitting to the data. Altogether, these data indicated that (i) this technology was suitable for our study and (ii) that fluorescence quenching is appropriate for determining the K_D_ between MuRF1 and E2 enzymes.

#### 3.3.2. MuRF1 Exhibits a Better Affinity for Filamentous Than for Monomeric Actin

As a second step, we addressed the affinity constants between MuRF1 and a-actin or myosin, which has never been addressed before. Indeed, using immunoprecipitation and yeast two-hybrid approaches, we and others found that MuRF1 strongly interacts with a-actin [9] and with MHCI and MHCIIa [7]. However, both techniques do not allow for the quantification of affinity between two proteins.

**Figure 4:**
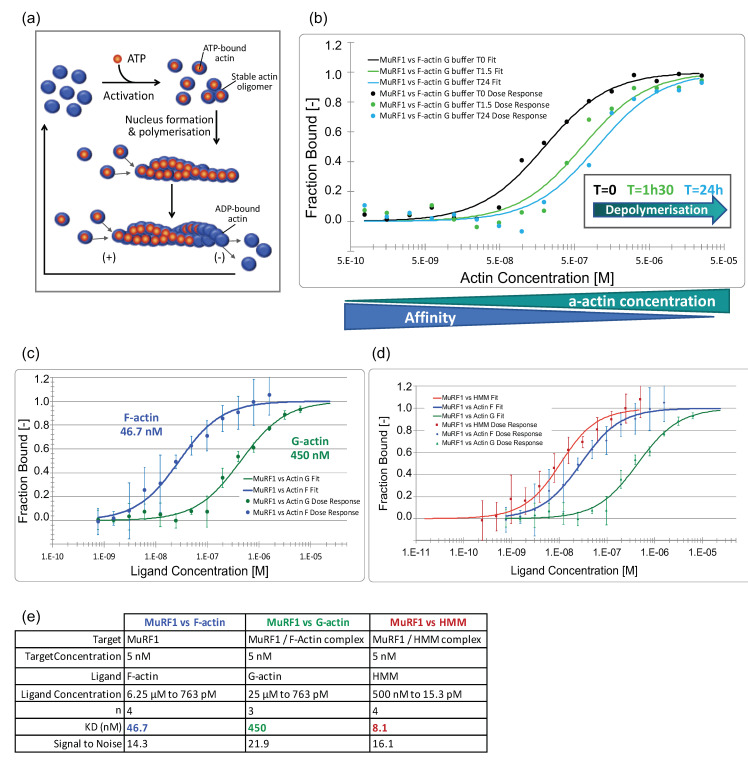
MuRF1 has a high affinity for filamentous actin and heavy meromyosin. (**a**) Scheme of actin poly- and depolymerization. Actin can be present either as a free monomer (G-actin) or as a linear polymer microfilament called F-actin (filamentous). Monomeric actin polymerizes at >2 mM K+ or Na+, and at >0.05 mM Mg2+. The (+) end of the actin filament tends to capture ATP-actin, thus promoting polarization, while, at the (−) end, the actin filament is mostly composed of ADP-actin that promotes depolymerization. A stationary equilibrium is achieved when the G-actin monomers are exchanged at both ends of the microfilament without any change to its total length. (**b**) Interaction of MuRF1 with F-a-actin during F-a-actin depolymerization. Capillaries containing MuRF1 and F-actin in G buffer were read immediately (T = 0 min) or stored for 1 h 30 or 24 h before reading, leading to the depolymerization of actin). (**c**) Dose–response curves for MuRF1 vs. F-actin in F buffer and vs. G-actin in G buffer. (**d**) Dose–response curves for MuRF 1 vs. F-actin in F buffer, vs. G-actin in G buffer and vs. heavy meromyosin (HMM, HMM includes the first 800 amino acids of the MHC, plus the two pairs of light chains). (**e**) Dataset overview.

Actin can exist as a free monomer called globular actin (G-actin) or as part of filamentous polymer called F-actin (Figure 4a and Supplementary Materials Figure S4). According to the buffer used, a-actin can be kept under its monomeric G-actin form or “pushed” toward filamentous F-actin polymerization. In muscle cells, a-actin also exists in both forms, with the filamentous form being part of the contractile apparatus. To figure out whether MuRF1 behaves differently depending on the state of actin, monomeric vs. filamentous form, we incubated MuRF1 with F-actin and induced its depolymerization by diluting F-buffer to reconstitute the G buffer (Figure 4b and Supplementary Materials Figure S4). Capillaries containing MuRF1 and F-actin in G buffer were read immediately (T = 0 min) and then read after 1 h 30 and 24 h of incubation in the dark, at 22 °C, to let F-actin depolymerization to occur. The graph in Figure 4b shows that MuRF1–actin interaction actually occurred in our system. More importantly, depolymerization of F-actin induced a shift of the curve to the right, indicating a decrease in affinity. To validate this result, we measured the affinity between MuRF1 and F-actin in F-buffer to maintain a-actin in a filamentous form and the affinity between MuRF1 and G-actin in G-buffer to maintain a-actin in monomeric form (Figure 4c,d). We confirmed that MuRF1 exhibits a better affinity for filamentous (46.7 nM) than for monomeric actin (450 nM). These data support our previous results indicating that MuRF1 preferentially targets sarcomeric a-actin than the soluble cytosolic form [9].

To determine the affinity between MuRF1 and myosin, we used the soluble part of myosin, heavy meromyosin (HMM), purified from rabbit skeletal muscle (Cytoskeleton, Inc., Denver, CO, USA). HMM includes the first 800 of the approximately 2000 residues of the MHC plus the two pairs of light chains (essential light chain and regulatory light chain). Indeed, MuRF1 has been shown to interact with MHCI and MHCIIa via their N-terminal head domain [7]. We confirmed that MuRF1 strongly interacts with HMM in our system and determined that the affinity constant was in the nanomolar range (8 nM) (Figure 4d,e and Supplementary Materials Figure S4).

#### 3.3.3. The Affinity between MuRF1 and E2L3 Is Not Enhanced by Actin or Myosin

**Figure 5:**
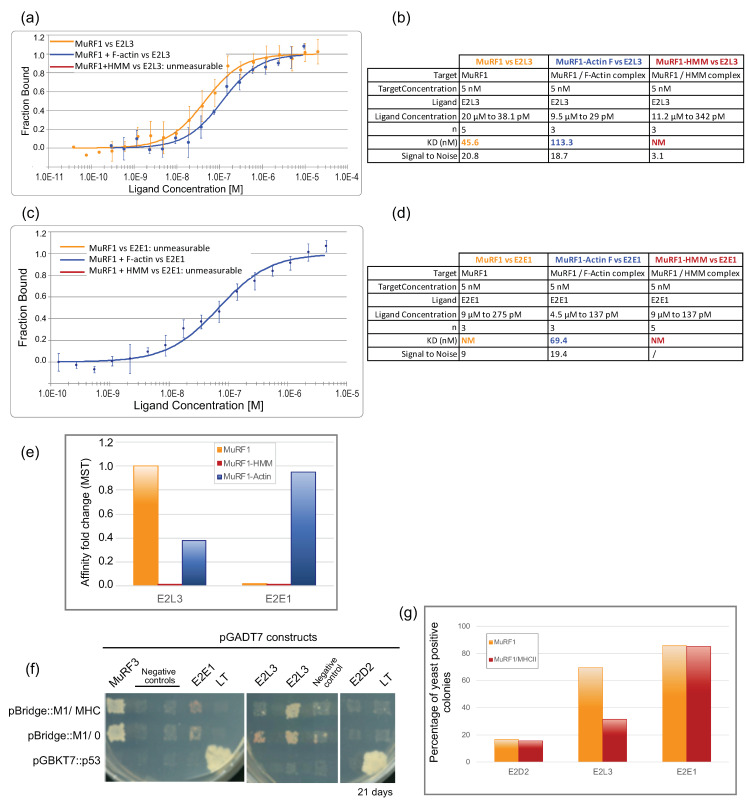
Impact of substrates on MuRF1–E2 interaction. (**a**) Dose–response curves for MuRF1–E2L3 interaction in presence or absence of substrates, a-actin or HMM (myosin). (**b**) Dataset overview of the photobleaching experiments performed with E2L3 in (**a**). (**c**) Dose–response curves for MuRF1–E2E1 interaction in presence or absence of substrates. (**d**) Dataset overview of the photobleaching experiment performed with E2E1 in (**c**). (**e**) Affinity change based on data presented in (**a**–**d**). (**f**) Impact of MHCIIa (residues 1–255) on the interaction between MuRF1 and E2L3, E2E1 or E2D2, determined by yeast-three-hybrid (Y3H) experiments. Diploid yeast colonies were replicated on selection plates (medium lacking leucine, tryptophan and histidine and supplemented with 20 mM Aureobasidin A and 2.5 mM 3-Amino-1,2,4-triazole), allowing the readout of interaction occurrence. Four independent transformations were performed, and 10 to 16 colonies were analyzed for each interaction studied. The strong interactions MuRF1–MuRF3 and p53–Large-T served as positive controls. Representative results are shown; two different “E2L3 + MuRF1/MHC” clones are presented to illustrate that MHC impacted the growth of 50% of the clones in contrast to the “E2L3 +MuRF1” clones. (**g**) Data from Y3H experiments were presented as percentage of clones that have grown on selective media (see Section 2 for details). Ten to 16 clones were analyzed for each interaction.

We then investigated whether F-actin and MHC could modulate the interaction between MuRF1 and the E2 enzyme, as it was the case between MuRF1 and some E2s in the presence of telethonin [23]. Then 5 nM of fluorescent MuRF1 was incubated with five times more moles of substrate, F-actin or HMM, at 22 °C during 30 min, to allow complexes formation and stabilization, followed by the addition of increasing concentrations of the E2 enzyme (E2L3 or E2E1) (Figure 5a–e and Supplementary Materials Figure S5).

The presence of F-a-actin (Figure 5b, blue curve) did not improve the interaction between MuRF1 and E2L3. We observed a slight shift of the K_D_ from 45 nM with MuRF1 alone to 113 nM with MuRF1 complexed to F-a-actin, meaning that the affinity was similar in both conditions, with even a tendency to be lower in the presence of F-a-actin (2.5 fold) (Figure 5e). Surprisingly, the presence of myosin complexed to MuRF1 negatively impacted the interaction between MuRF1 and E2L3, which could no longer be analyzed in these conditions (absence of a red fit in Figure 5a), despite the use of high concentrations of E2L3 up to 11.2 μM (Figure 5b). This indicated that myosin prevented MuRF1–E2L3 interaction. However, the negative impact of myosin on MuRF1–E2 interaction could also be due to the in vitro context (e.g., use of recombinant proteins and unloaded E2 enzymes, absence of cellular environment, post-translational modifications, etc.). Regarding E2E1, the presence of F-a-actin greatly improved and stabilized the interaction between MuRF1 and E2E1 (Figure 5c), which exhibited a high affinity (K_D_ = 69 nM). By contrast, the presence of myosin did not stabilize the interaction between MuRF1 and E2E1 in vitro.

We then moved to a yeast-three-hybrid (Y3H) approach to better understand the role of myosin on the MuRF1–E2L3 interaction in a eukaryotic context, where (i) proteins are more likely in their native conformation and (ii) the UPS machinery is present, e.g., if Ub loading on E2 enzymes is necessary for the interaction. Since a-actin and full-length MHC cannot be handled in Y3H due to their tendency to polymerize and/or aggregate, we focused only on the N-terminal part of MHCIIa that was previously shown to interact with MuRF1 (the first 255 residues) [7]. Co-expression of MuRF1 and Large-T protein was set as the background level (Figure 5f). The co-expression of MuRF1 and MuRF3 assessed the correct expression and folding of MuRF1 (Figure 5f). Yeasts co-expressing E2D2 and MuRF1 ± MHCIIa (1–255) grew poorly on selective medium. Less than 17% of the clones tested were able to grow in three weeks, revealing a lack of interaction between E2D2 and MuRF1 (~17% for MuRF1 alone, ~16% for MuRF1/MHCIIa). These data support a previous report indicating that E2D2 is not involved in MuRF1-dependent muscle wasting during hind-limb suspension [51]. Regarding E2L3, ~70% of the diploid clones co-expressing E2L3 and MuRF1 grew on selective media, as previously published [23]. This percentage dropped to ~32% if the yeast co-expressed MHCIIa with MuRF1 (Figure 5g), indicating that myosin interfered with the interaction between MuRF1 and E2L3, thus corroborating the data obtained with the Monolith. Regarding E2E1, the presence of myosin did not modify the percentage of positive colonies (around 80%), suggesting that myosin did not prevent MuRF1–E2E1 interaction to occur. These data suggested that either something was previously missing in the in vitro Monolith assay (for example Ub loaded on the E2) or that myosin did not stabilize MuRF1–E2E1 enough to be visible with the Monolith assays.

## 4. Discussion

We previously showed that the ubiquitin-conjugating E2 enzyme E2L3 can interact with MuRF1/TRIM63, a ubiquitin E3 ligase involved in skeletal muscle atrophy during catabolic conditions [23]. MuRF1 is the only E3 ligase known to target the main contractile proteins for degradation [7,8,9,20]. As for most E2 enzymes, the role of E2L3/UbcH7 is yet unknown in skeletal muscle. Here, we report, for the first time, a link between E2L3 and the degradation of the main constituents of the contractile apparatus, a-actin and myosin in catabolic C2C12 myotubes. We also report that in vivo E2L3 may be involved in muscle atrophy processes. Unexpectedly, the implication of E2L3 on myofibrillar protein degradation may be at least partly independent of MuRF1.

Inhibiting muscle atrophy has been shown to extend the life span of tumor bearing animals [52]. Developing such strategies has thus raised the interest of several laboratories for targeting different actors of muscle atrophy (for reviews, see References [13,53]). In this field, MuRF1 is of particular interest, since MuRF1 targets the main contractile proteins for degradation during catabolic states (for review, see Reference [21]). However, strict inhibition of MuRF1 may induce side effects due to its pleiotropic functions [21]. Targeting E2 enzymes would be worse because of the limited number of E2 enzymes present in the cell. Not surprisingly, mutations of some E2s (including E2L3) have been associated with different diseases and cancer [22]. Thus, targeting the interface between MuRF1 and the E2(s) specifically involved in the degradation of contractile proteins rather than individual enzymes, may represent attractive therapeutic strategies to fight against muscle atrophy. Interestingly, E2L3 is highly expressed in skeletal muscles and exhibits a high affinity against MuRF1 [23].

Knockdown experiments (Figure 1) revealed that E2L3 silencing significantly preserved a-actin and myosin (MHCIIa) levels in myotubes, thus suggesting an important role of E2L3 on contractile protein degradation. The KD and overexpression of E2L3 in T. anterior muscles of mice further confirmed the results obtained in C2C12 myotubes. When overexpressed, E2L3 worsened muscle atrophy, whereas its KD led to hypertrophy of T. anterior in Dex-treated mice (Figure 2). By contrast, data from the literature showed that overexpression of MuRF1 was not sufficient for promoting muscle atrophy and MuRF1 KO was not able to promote hypertrophy (for a review, see Reference [21]). Altogether, E2L3 may have a greater impact than MuRF1 on skeletal muscle protein metabolism, which may implicate other E3 ligases.

We previously found that the targeting of the substrates by MuRF1–E2 was more complex than expected and that the presence of a substrate was able to allosterically modify the affinity between MuRF1 and the E2 enzymes [23]. Indeed, the presence of telehonin allosterically strengthened and stabilized MuRF1–E2E1 and MuRF1–E2J1 interactions [23]. To determine whether this mechanism is widely present for MuRF1 substrates, we thus addressed the affinity and stability of MuRF1–E2L3 couple in presence of a-actin or myosin. The interactions between MuRF1 and its substrates a-actin and MHC were shown by using several approaches (yeast hybrid systems, pulldown, etc.) (for review see, Reference [21]). However, the particular properties of a-actin and MHC that tend to polymerize and/or aggregate in vitro impeded any determination of their binding constant. Here, we report, for the first time, that MuRF1 exhibits a strong affinity for filamentous a-actin and the soluble portion of myosin (Heavy Meromyosin, HMM) (K_D_ = 46.7 nM and 8 nM respectively). Remarkably, MuRF1 showed a better affinity for filamentous than for monomeric actin (Figure 4). This suggests that MuRF1 may be part of the depolymerization process of a-actin in atrophying conditions. These data are consistent with other reports suggesting the release of actomyosin fragments from the myofibrillar structure. Indeed, MuRF1 was shown to target myofibrillar a-actin for degradation upon Dex treatment [9]. Moreover, data from Pr Goldberg’s laboratory have demonstrated that desmin intermediate filaments depolymerization is an early event during atrophy. Desmin depolymerization preceded the release of myofilaments from the myofibril by the ubiquitin-dependent p97/VCP ATPase complex, thus allowing proteasomal degradation of myofibrillar proteins [54,55]. Importantly, the targeting of thick filaments was MuRF1-dependent, as confirmed by their protection in muscles lacking functional MuRF1 [10].

The tripartite interaction assays we performed suggest that substrates behave differently. Combined with already published results [23,45], these data suggest that differential interactions prevail with MuRF1, depending on both the substrate and the E2: telethonin and a-actin allosterically favor E2E1 or E2J1 [23], while MHC impedes E2E1 and E2L3 binding (Figure 5). Consistently, MuRF1–E2E1 is able to target for degradation telethonin [23] and a-actin [45] but not MHCIIa in HEK293T cells [45]. Moreover, Fielitz and collaborators reported that, in in vitro ubiquitination assays, MuRF1 was unable to polyubiquitinate MHCIIa and MHCI, using either E2E1 or E2L3 [7], which is consistent with our results. Nonetheless, the substrate-dependent allosteric stabilization of some E2 enzymes (E2E1 and EJ1) on MuRF1 warrants further investigations to decipher the physiological significance of this adaptation. Altogether, the MuRF1–E2 network may be specialized for dedicated roles during the degradation of skeletal muscle proteins, with each E2 being used by MuRF1 for specific purposes. Future investigations will have to precisely decipher the role of each E2 and their potential hierarchy in MuRF1-driven sarcomeric proteins targeting.

We demonstrated here that E2L3 plays an important role in contractile proteins degradation that may be MuRF1-dependent and/or -independent. Indeed, it is noteworthy that E2L3 interacts with other E3 ligases, such as LUBAC [56,57], c-CBL [28,58,59] and PARKIN [60,61,62], that have been involved in muscle-mass control [13]. For example, in skeletal muscle, cachexia is characterized by inflammation that contributes to muscle atrophy through the stimulation of proteolysis via nuclear factor κB (NF-κB) pathway [13]. LUBAC, in cooperation with E2L3, synthesizes Met1-linked ubiquitin chains to NEMO, resulting in IKK activation and subsequent activation of the NF-κB signaling pathway [56,63]. MuRF1 is controlled by several signaling pathways, including NF-κB, which suggests that E2L3 may be implicated both directly and indirectly in muscle-atrophy processes. Future studies will have to determine the exact implication of E2L3 with its different E3 ligase partners in catabolic skeletal muscle.

## 5. Conclusions

Preventing muscle wasting represents a promising strategy for improving patients’ health and therapeutic treatments’ efficiency. Combined with the literature, our data show that both MuRF1 and E2L3 are important regulators of muscle mass, potentially acting via several mechanisms. However, the exact role of each partner remains to be established, as the presence of the substrate adds complexity to the mechanisms involved. Whether each substrate is handled by a dedicated MuRF1–E2 couple remains to be established, and future studies will have to confirm this hypothesis.

## Supplementary Materials

The following are available online at https://www.mdpi.com/article/10.3390/cells10081974/s1, Figure S1: Control of MuRF1 and E2L3 silencing in C2C12 myotubes. Figure S2: Transfection efficiency of shRNA in Tibialis anterior muscles. Figures S3–S5: MST experiments control (MST-Traces Chart and Capillary shape) for Figures 3–5, respectively. Table S1: List of sequences of shRNAs targeting E2L3.

## References

[1] Von Haehling S., Anker S.D. (2010). Cachexia as a Major Underestimated and Unmet Medical Need: Facts and Numbers. J. Cachexia Sarcopenia Muscle.

[2] Von Haehling S., Anker M.S., Anker S.D. (2016). Prevalence and Clinical Impact of Cachexia in Chronic Illness in Europe, USA, and Japan: Facts and Numbers Update 2016. J. Cachexia Sarcopenia Muscle.

[3] Furuno K., Goodman M.N., Goldberg A.L. (1990). Role of Different Proteolytic Systems in the Degradation of Muscle Proteins during Denervation Atrophy. J. Biol. Chem..

[4] Solomon V., Goldberg A.L. (1996). Importance of the ATP-Ubiquitin-Proteasome Pathway in the Degradation of Soluble and Myofibrillar Proteins in Rabbit Muscle Extracts. J. Biol. Chem..

[5] Tawa N.E., Odessey R., Goldberg A.L. (1997). Inhibitors of the Proteasome Reduce the Accelerated Proteolysis in Atrophying Rat Skeletal Muscles. J. Clin. Investig..

[6] Ventadour S., Attaix D. (2006). Mechanisms of Skeletal Muscle Atrophy. Curr. Opin. Rheumatol..

[7] Fielitz J., Kim M.-S., Shelton J.M., Latif S., Spencer J.A., Glass D.J., Richardson J.A., Bassel-Duby R., Olson E.N. (2007). Myosin Accumulation and Striated Muscle Myopathy Result from the Loss of Muscle RING Finger 1 and 3. J. Clin. Investig..

[8] Clarke B.A., Drujan D., Willis M.S., Murphy L.O., Corpina R.A., Burova E., Rakhilin S.V., Stitt T.N., Patterson C., Latres E. (2007). The E3 Ligase MuRF1 Degrades Myosin Heavy Chain Protein in Dexamethasone-Treated Skeletal Muscle. Cell Metab..

[9] Polge C., Heng A.-E., Jarzaguet M., Ventadour S., Claustre A., Combaret L., Bechet D., Matondo M., Uttenweiler-Joseph S., Monsarrat B. (2011). Muscle Actin Is Polyubiquitinylated In Vitro and In Vivo and Targeted for Breakdown by the E3 Ligase MuRF1. FASEB J..

[10] Cohen S., Brault J.J., Gygi S.P., Glass D.J., Valenzuela D.M., Gartner C., Latres E., Goldberg A.L. (2009). During Muscle Atrophy, Thick, but Not Thin, Filament Components Are Degraded by MuRF1-Dependent Ubiquitylation. J. Cell Biol..

[11] Ikeda F., Crosetto N., Dikic I. (2010). What Determines the Specificity and Outcomes of Ubiquitin Signaling?. Cell.

[12] Stewart M.D., Ritterhoff T., Klevit R.E., Brzovic P.S. (2016). E2 Enzymes: More than Just Middle Men. Cell Res..

[13] Peris-Moreno D., Cussonneau L., Combaret L., Polge C., Taillandier D. (2021). Ubiquitin Ligases at the Heart of Skeletal Muscle Atrophy Control. Molecules.

[14] Taillandier D., Polge C. (2019). Skeletal Muscle Atrogenes: From Rodent Models to Human Pathologies. Biochimie.

[15] Lecker S.H., Jagoe R.T., Gilbert A., Gomes M., Baracos V., Bailey J., Price S.R., Mitch W.E., Goldberg A.L. (2004). Multiple Types of Skeletal Muscle Atrophy Involve a Common Program of Changes in Gene Expression. FASEB J..

[16] Bodine S.C., Latres E., Baumhueter S., Lai V.K., Nunez L., Clarke B.A., Poueymirou W.T., Panaro F.J., Na E., Dharmarajan K. (2001). Identification of Ubiquitin Ligases Required for Skeletal Muscle Atrophy. Science.

[17] Labeit S., Kohl C.H., Witt C.C., Labeit D., Jung J., Granzier H. (2010). Modulation of Muscle Atrophy, Fatigue and MLC Phosphorylation by MuRF1 as Indicated by Hindlimb Suspension Studies on MuRF1-KO Mice. J. Biomed. Biotechnol..

[18] Baehr L.M., Furlow J.D., Bodine S.C. (2011). Muscle Sparing in Muscle RING Finger 1 Null Mice: Response to Synthetic Glucocorticoids. J. Physiol..

[19] Vann C.G., Roberson P.A., Osburn S.C., Mumford P.W., Romero M.A., Fox C.D., Moore J.H., Haun C.T., Beck D.T., Moon J.R. (2020). Skeletal Muscle Myofibrillar Protein Abundance Is Higher in Resistance-Trained Men, and Aging in the Absence of Training May Have an Opposite Effect. Sports.

[20] Kedar V., McDonough H., Arya R., Li H.-H., Rockman H.A., Patterson C. (2004). Muscle-Specific RING Finger 1 Is a Bona Fide Ubiquitin Ligase That Degrades Cardiac Troponin I. Proc. Natl. Acad. Sci. USA.

[21] Peris-Moreno D., Taillandier D., Polge C. (2020). MuRF1/TRIM63, Master Regulator of Muscle Mass. Int. J. Mol. Sci..

[22] Alpi A.F., Chaugule V., Walden H. (2016). Mechanism and Disease Association of E2-Conjugating Enzymes: Lessons from UBE2T and UBE2L3. Biochem. J..

[23] Polge C., Cabantous S., Deval C., Claustre A., Hauvette A., Bouchenot C., Aniort J., Béchet D., Combaret L., Attaix D. (2018). A Muscle-Specific MuRF1-E2 Network Requires Stabilization of MuRF1-E2 Complexes by Telethonin, a Newly Identified Substrate. J. Cachexia Sarcopenia Muscle.

[24] Deval C., Calonne J., Coudy-Gandilhon C., Vazeille E., Bechet D., Polge C., Taillandier D., Attaix D., Combaret L. (2020). Mitophagy and Mitochondria Biogenesis Are Differentially Induced in Rat Skeletal Muscles during Immobilization and/or Remobilization. Int. J. Mol. Sci..

[25] Wenzel D.M., Lissounov A., Brzovic P.S., Klevit R.E. (2011). UBCH7 Reactivity Profile Reveals Parkin and HHARI to Be RING/HECT Hybrids. Nature.

[26] Soss S.E., Yue Y., Dhe-Paganon S., Chazin W.J. (2011). E2 Conjugating Enzyme Selectivity and Requirements for Function of the E3 Ubiquitin Ligase CHIP*. J. Biol. Chem..

[27] Zheng N., Wang P., Jeffrey P.D., Pavletich N.P. (2000). Structure of a C-Cbl-UbcH7 Complex: RING Domain Function in Ubiquitin-Protein Ligases. Cell.

[28] Yokouchi M., Kondo T., Houghton A., Bartkiewicz M., Horne W.C., Zhang H., Yoshimura A., Baron R. (1999). Ligand-Induced Ubiquitination of the Epidermal Growth Factor Receptor Involves the Interaction of the c-Cbl RING Finger and UbcH7*. J. Biol. Chem..

[29] Nakao R., Hirasaka K., Goto J., Ishidoh K., Yamada C., Ohno A., Okumura Y., Nonaka I., Yasutomo K., Baldwin K.M. (2009). Ubiquitin Ligase Cbl-b Is a Negative Regulator for Insulin-Like Growth Factor 1 Signaling during Muscle Atrophy Caused by Unloading. Mol. Cell. Biol..

[30] Ahel J., Lehner A., Vogel A., Schleiffer A., Meinhart A., Haselbach D., Clausen T. (2020). Moyamoya Disease Factor RNF213 Is a Giant E3 Ligase with a Dynein-like Core and a Distinct Ubiquitin-Transfer Mechanism. eLife.

[31] Pao K.-C., Wood N.T., Knebel A., Rafie K., Stanley M., Mabbitt P.D., Sundaramoorthy R., Hofmann K., van Aalten D.M.F., Virdee S. (2018). Activity-Based E3 Ligase Profiling Uncovers an E3 Ligase with Esterification Activity. Nature.

[32] Pao K.-C., Stanley M., Han C., Lai Y.-C., Murphy P., Balk K., Wood N.T., Corti O., Corvol J.-C., Muqit M.M.K. (2016). Probes of Ubiquitin E3 Ligases Enable Systematic Dissection of Parkin Activation. Nat. Chem. Biol.

[33] Han J.-W., Zheng H.-F., Cui Y., Sun L.-D., Ye D.-Q., Hu Z., Xu J.-H., Cai Z.-M., Huang W., Zhao G.-P. (2009). Genome-Wide Association Study in a Chinese Han Population Identifies Nine New Susceptibility Loci for Systemic Lupus Erythematosus. Nat. Genet..

[34] Wang S., Adrianto I., Wiley G.B., Lessard C.J., Kelly J.A., Adler A.J., Glenn S.B., Williams A.H., Ziegler J.T., Comeau M.E. (2012). A Functional Haplotype of UBE2L3 Confers Risk for Systemic Lupus Erythematosus. Genes Immun..

[35] Kim T., Bae S.-C., Kang C. (2020). Synergistic Activation of NF-ΚB by TNFAIP3 (A20) Reduction and UBE2L3 (UBCH7) Augment That Synergistically Elevate Lupus Risk. Arthritis Res. Ther..

[36] Franke A., McGovern D.P.B., Barrett J.C., Wang K., Radford-Smith G.L., Ahmad T., Lees C.W., Balschun T., Lee J., Roberts R. (2010). Genome-Wide Meta-Analysis Increases to 71 the Number of Confirmed Crohn’s Disease Susceptibility Loci. Nat. Genet..

[37] Tsoi L.C., Spain S.L., Knight J., Ellinghaus E., Stuart P.E., Capon F., Ding J., Li Y., Tejasvi T., Gudjonsson J.E. (2012). Identification of 15 New Psoriasis Susceptibility Loci Highlights the Role of Innate Immunity. Nat. Genet..

[38] Whitcomb E.A., Dudek E.J., Liu Q., Taylor A. (2008). Novel Control of S Phase of the Cell Cycle by Ubiquitin-Conjugating Enzyme H7. MBoC.

[39] Whitcomb E.A., Taylor A. (2009). Ubiquitin Control of S Phase: A New Role for the Ubiquitin Conjugating Enzyme, UbcH7. Cell Div..

[40] Whitcomb E.A., Tsai Y.C., Basappa J., Liu K., le Feuvre A.K., Weissman A.M., Taylor A. (2019). Stabilization of P27Kip1/CDKN1B by UBCH7/UBE2L3 Catalyzed Ubiquitinylation: A New Paradigm in Cell-Cycle Control. FASEB J..

[41] Oh K.-J., Kalinina A., Wang J., Nakayama K., Nakayama K.I., Bagchi S. (2004). The Papillomavirus E7 Oncoprotein Is Ubiquitinated by UbcH7 and Cullin 1- and Skp2-Containing E3 Ligase. J. Virol..

[42] Anoveros-Barrera A., Bhullar A.S., Stretch C., Dunichand-Hoedl A.R., Martins K.J.B., Rieger A., Bigam D., McMullen T., Bathe O.F., Putman C.T. (2019). Immunohistochemical Phenotyping of T Cells, Granulocytes, and Phagocytes in the Muscle of Cancer Patients: Association with Radiologically Defined Muscle Mass and Gene Expression. Skelet. Muscle.

[43] Han X., Zhang L., Chung J., Pozo F.M., Tran A., Seachrist D.D., Jacobberger J.W., Keri R.A., Gilmore H., Zhang Y. (2014). UbcH7 Regulates 53BP1 Stability and DSB Repair. Proc. Natl. Acad. Sci. USA.

[44] Mayca Pozo F., Tang J., Bonk K.W., Keri R.A., Yao X., Zhang Y. (2017). Regulatory Cross-Talk Determines the Cellular Levels of 53BP1 Protein, a Critical Factor in DNA Repair. J. Biol. Chem..

[45] Devine M.J., Plun-Favreau H., Wood N.W. (2011). Parkinson’s Disease and Cancer: Two Wars, One Front. Nat. Rev. Cancer.

[46] Polge C., Aniort J., Armani A., Claustre A., Coudy-Gandilhon C., Tournebize C., Deval C., Combaret L., Béchet D., Sandri M. (2018). UBE2E1 Is Preferentially Expressed in the Cytoplasm of Slow-Twitch Fibers and Protects Skeletal Muscles from Exacerbated Atrophy upon Dexamethasone Treatment. Cells.

[47] Soares R.J., Cagnin S., Chemello F., Silvestrin M., Musaro A., De Pitta C., Lanfranchi G., Sandri M. (2014). Involvement of MicroRNAs in the Regulation of Muscle Wasting during Catabolic Conditions*. J. Biol. Chem..

[48] Wienken C.J., Baaske P., Rothbauer U., Braun D., Duhr S. (2010). Protein-Binding Assays in Biological Liquids Using Microscale Thermophoresis. Nat. Commun.

[49] Jerabek-Willemsen M., Wienken C.J., Braun D., Baaske P., Duhr S. (2011). Molecular Interaction Studies Using Microscale Thermophoresis. ASSAY Drug Dev. Technol..

[50] Polge C., Koulmann N., Claustre A., Jarzaguet M., Serrurier B., Combaret L., Béchet D., Bigard X., Attaix D., Taillandier D. (2016). UBE2D2 Is Not Involved in MuRF1-Dependent Muscle Wasting during Hindlimb Suspension. Int. J. Biochem. Cell Biol..

[51] Zhou X., Wang J.L., Lu J., Song Y., Kwak K.S., Jiao Q., Rosenfeld R., Chen Q., Boone T., Simonet W.S. (2010). Reversal of Cancer Cachexia and Muscle Wasting by ActRIIB Antagonism Leads to Prolonged Survival. Cell.

[52] Scalabrin M., Adams V., Labeit S., Bowen T.S. (2020). Emerging Strategies Targeting Catabolic Muscle Stress Relief. Int. J. Mol. Sci..

[53] Piccirillo R., Goldberg A.L. (2012). The P97/VCP ATPase Is Critical in Muscle Atrophy and the Accelerated Degradation of Muscle Proteins. EMBO J..

[54] Volodin A., Kosti I., Goldberg A.L., Cohen S. (2017). Myofibril Breakdown during Atrophy Is a Delayed Response Requiring the Transcription Factor PAX4 and Desmin Depolymerization. Proc. Natl. Acad. Sci. USA.

[55] Tokunaga F., Sakata S., Saeki Y., Satomi Y., Kirisako T., Kamei K., Nakagawa T., Kato M., Murata S., Yamaoka S. (2009). Involvement of Linear Polyubiquitylation of NEMO in NF-KappaB Activation. Nat. Cell Biol.

[56] Stieglitz B., Rana R.R., Koliopoulos M.G., Morris-Davies A.C., Schaeffer V., Christodoulou E., Howell S., Brown N.R., Dikic I., Rittinger K. (2013). Structural Basis for Ligase-Specific Conjugation of Linear Ubiquitin Chains by HOIP. Nature.

[57] Nikawa T., Ishidoh K., Hirasaka K., Ishihara I., Ikemoto M., Kano M., Kominami E., Nonaka I., Ogawa T., Adams G.R. (2004). Skeletal Muscle Gene Expression in Space-Flown Rats. FASEB J..

[58] Uchida T., Sakashita Y., Kitahata K., Yamashita Y., Tomida C., Kimori Y., Komatsu A., Hirasaka K., Ohno A., Nakao R. (2018). Reactive Oxygen Species Upregulate Expression of Muscle Atrophy-Associated Ubiquitin Ligase Cbl-b in Rat L6 Skeletal Muscle Cells. Am. J. Physiol. Cell Physiol..

[59] Geisler S., Vollmer S., Golombek S., Kahle P.J. (2014). The Ubiquitin-Conjugating Enzymes UBE2N, UBE2L3 and UBE2D2/3 Are Essential for Parkin-Dependent Mitophagy. J. Cell Sci..

[60] Fiesel F.C., Moussaud-Lamodière E.L., Ando M., Springer W. (2014). A Specific Subset of E2 Ubiquitin-Conjugating Enzymes Regulate Parkin Activation and Mitophagy Differently. J. Cell Sci..

[61] Peker N., Donipadi V., Sharma M., McFarlane C., Kambadur R. (2018). Loss of Parkin Impairs Mitochondrial Function and Leads to Muscle Atrophy. Am. J. Physiol. Cell Physiol..

[62] Haas T.L., Emmerich C.H., Gerlach B., Schmukle A.C., Cordier S.M., Rieser E., Feltham R., Vince J., Warnken U., Wenger T. (2009). Recruitment of the Linear Ubiquitin Chain Assembly Complex Stabilizes the TNF-R1 Signaling Complex and Is Required for TNF-Mediated Gene Induction. Mol. Cell.

[63] Fujita H., Rahighi S., Akita M., Kato R., Sasaki Y., Wakatsuki S., Iwai K. (2014). Mechanism Underlying IκB Kinase Activation Mediated by the Linear Ubiquitin Chain Assembly Complex. Mol. Cell Biol..

