# Supplementary material for: UBE2L3, a Partner of MuRF1/TRIM63, Is Involved in the Degradation of Myofibrillar Actin and Myosin
Source: Cells. 2021 Aug 3;10(8):1974. doi: 10.3390/cells10081974 (PMC8392593; doi:10.3390/cells10081974)
Supplement: Supplementary file 1 [file cells-10-01974-s001.zip › cells-1185987-supplementary.pdf]

(a)

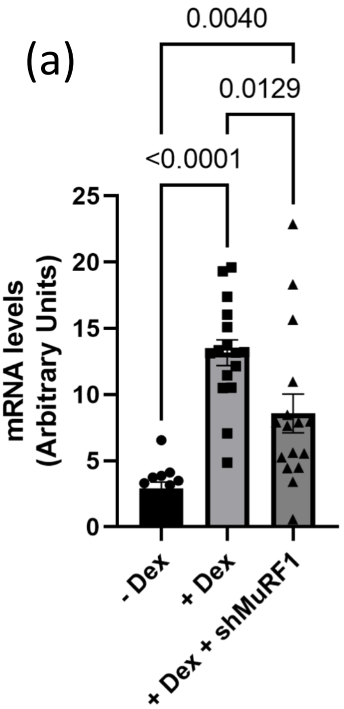

(b)

Genes	Sequences
**MuRF1**	5'- ATGGAGAACCTGGAGAAGCA -3'<br>5'- AACGACCTCCAGACATGGAC -3'
**YWHAZ**	5' – CTGGCCCTCAACTTCTCTGT – 3'<br>5' –AATGGCTTCATCGAAAGCTG – 3'
**Ppia**	5' – ACGCCACTGTCGCTTTTC – 3'<br>5' – CTGCAAACAGCTCGAAGGA – 3'

(c)

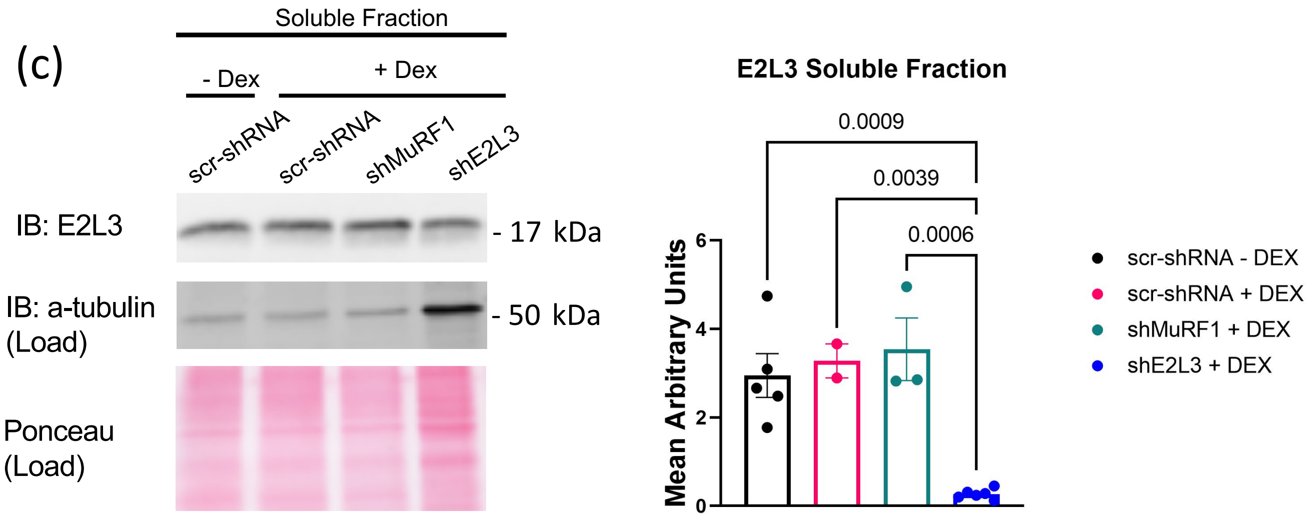

Figure S1. MuRF1 and E2L3 silencing in C2C12 myotubes. (**a**) mRNA levels of MuRF1. C2C12 myotubes were treated (+ Dex) or not (- Dex) with 1 µM Dex for 48 hours and electroporated as explained in the Methods section with two shRNA directed against MuRF1 (shMuRF1). One µM Dex induced an increase of MuRF1 mRNA levels, which was reversed by the shMuRF1. mRNA levels were determined by qRT-PCR. The reverse transcription of total RNA into DNA was performed using the QuantiTect Reverse Transcription kit (Qiagen, Venlo, The Netherlands). qPCR was performed using the FastStart DNA Master SYBR Green I kit (Roche, Basel, Switzerland), according to the manufacturer's instructions using a CFX96 thermocycler (BIORAD, Hercules, CA, USA). Calculations were made using the comparative DCt method with YWHAZ and HPRT1 housekeeping genes. (**b**) Oligos used for qRT-PCR. (**c**) Protein levels of E2L3. C2C12 myotubes were treated (+ Dex) or not (- Dex) with 1 µM Dex for 48 hours and electroporated as explained in the Methods section with two shRNA directed against E2L3 (shE2L3). Left panel, Western blot. Alpha-tubulin and Ponceau staining were used as loading controls and gave similar results. Right panel, densitometric analysis using a-tubulin for the normalization (see Methods section for details).

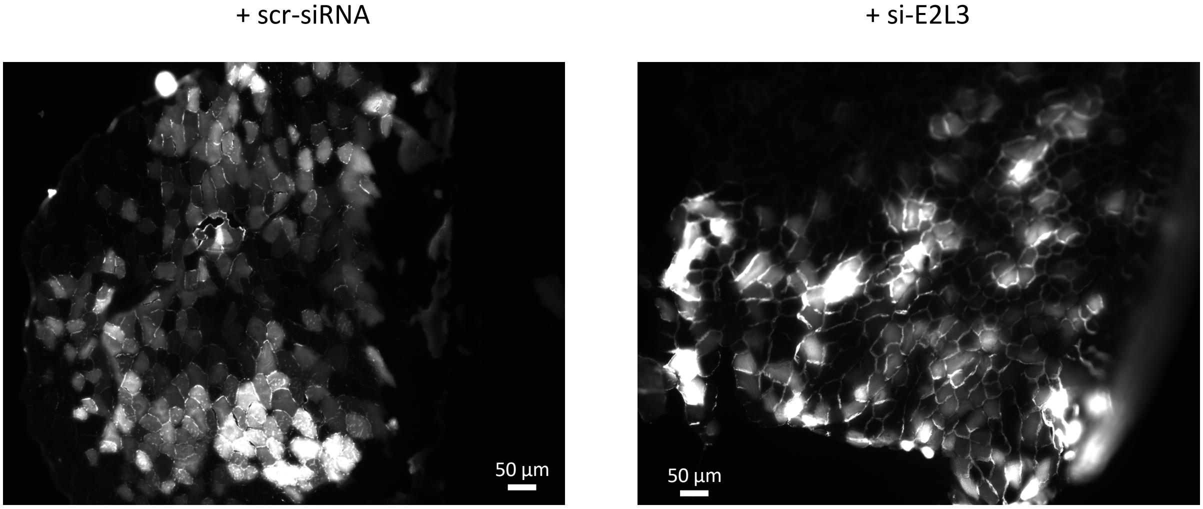

Figure S2. Transfection efficiency of shRNA in Tibialis anterior muscles. pCDNA 6.2 plasmids used for shRNA transfection also encode for emGFP allowing the visualization of transfected fibers. Typical transfection efficiency was around 50%.

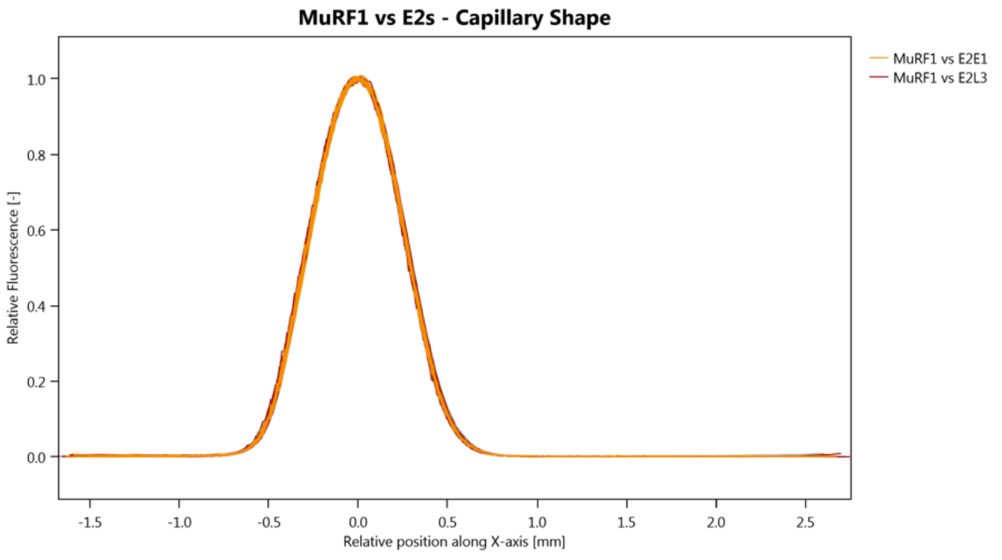

Figure S3. Additional data to Figure 3, MST experiments controls. Capillary shape MuRF1 vs E2E1 and E2L3. We verified that fluorescent NT647-MuRF1 was not adsorbed onto the 32 capillaries (16 for E2E1 and 16 for E2L3) as shown by the presence of the single peak and the perfect overlapping of the 32 curves.

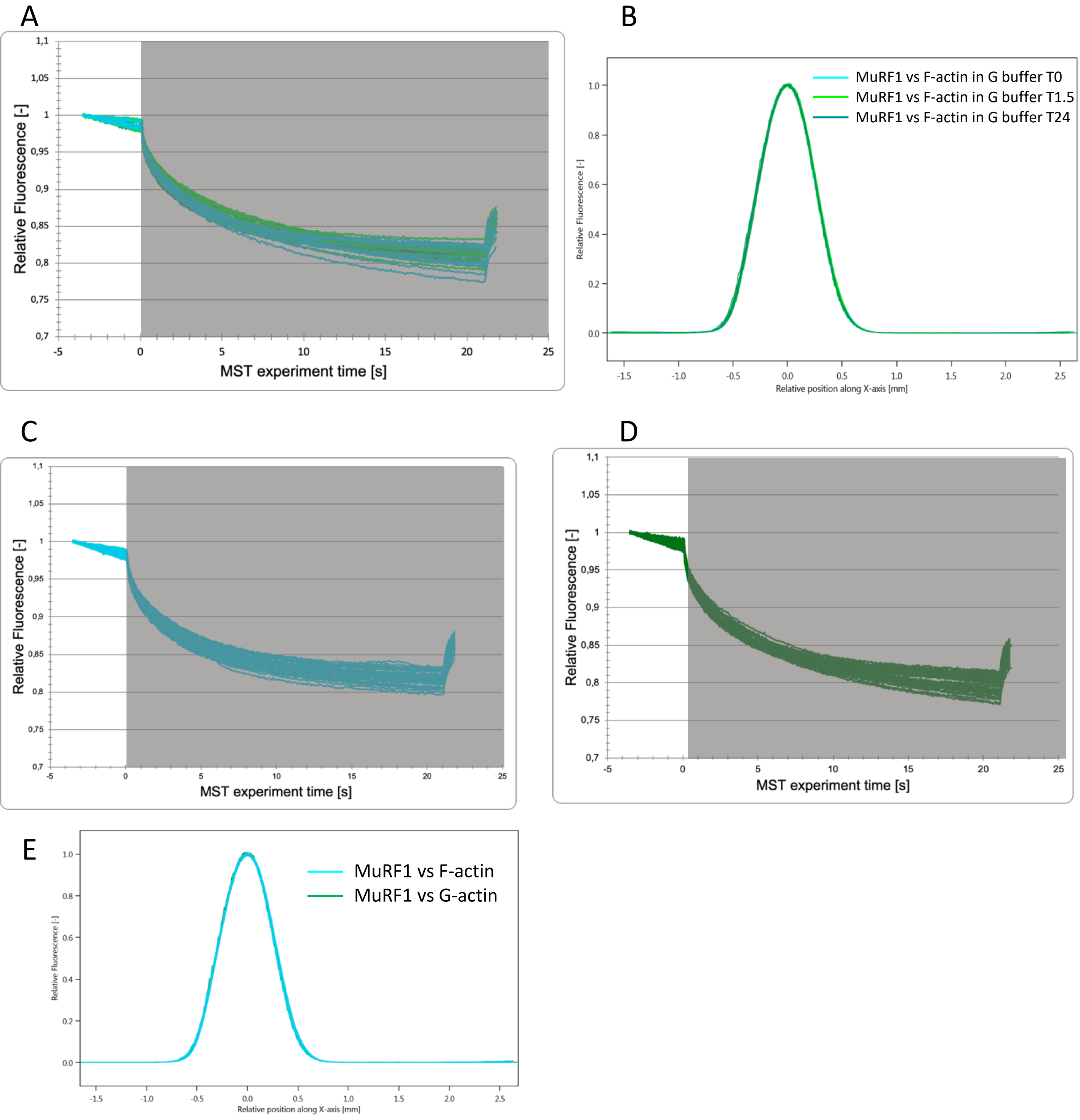

Figure S4. additional data to Figure 4b and 4c, MST experiments control

A. MST-Trace Chart for MuRF1 vs F-alpha-actin depolymerization (see legend of Figure 3 for details); supplement to Fig4b.
B. Capillary shape MuRF1 F-alpha-actin depolymerization . We verified that fluorescent NT647-MuRF1 was not adsorbed onto the capillaries as shown by the presence of the single peak and the perfect overlapping of the curves. supplement to Fig4b.
C. MST-Trace Chart for MuRF1 vs filamentous alpha-actin (see legend of Figure 3 for details); supplement to Fig4c.
D. MST-Trace Chart for MuRF1 vs monomeric alpha-actin (see legend of Figure 3 for details); supplement to Fig4c.
E. Capillary shape MuRF1 vs F- and G-alpha-actin. We verified that fluorescent NT647-MuRF1 was not adsorbed onto the 32 capillaries (16 for F-alpha-actin and 16 for G-alpha-actin) as shown by the presence of the single peak and the perfect overlapping of the 32 curves. supplement to Fig4c.

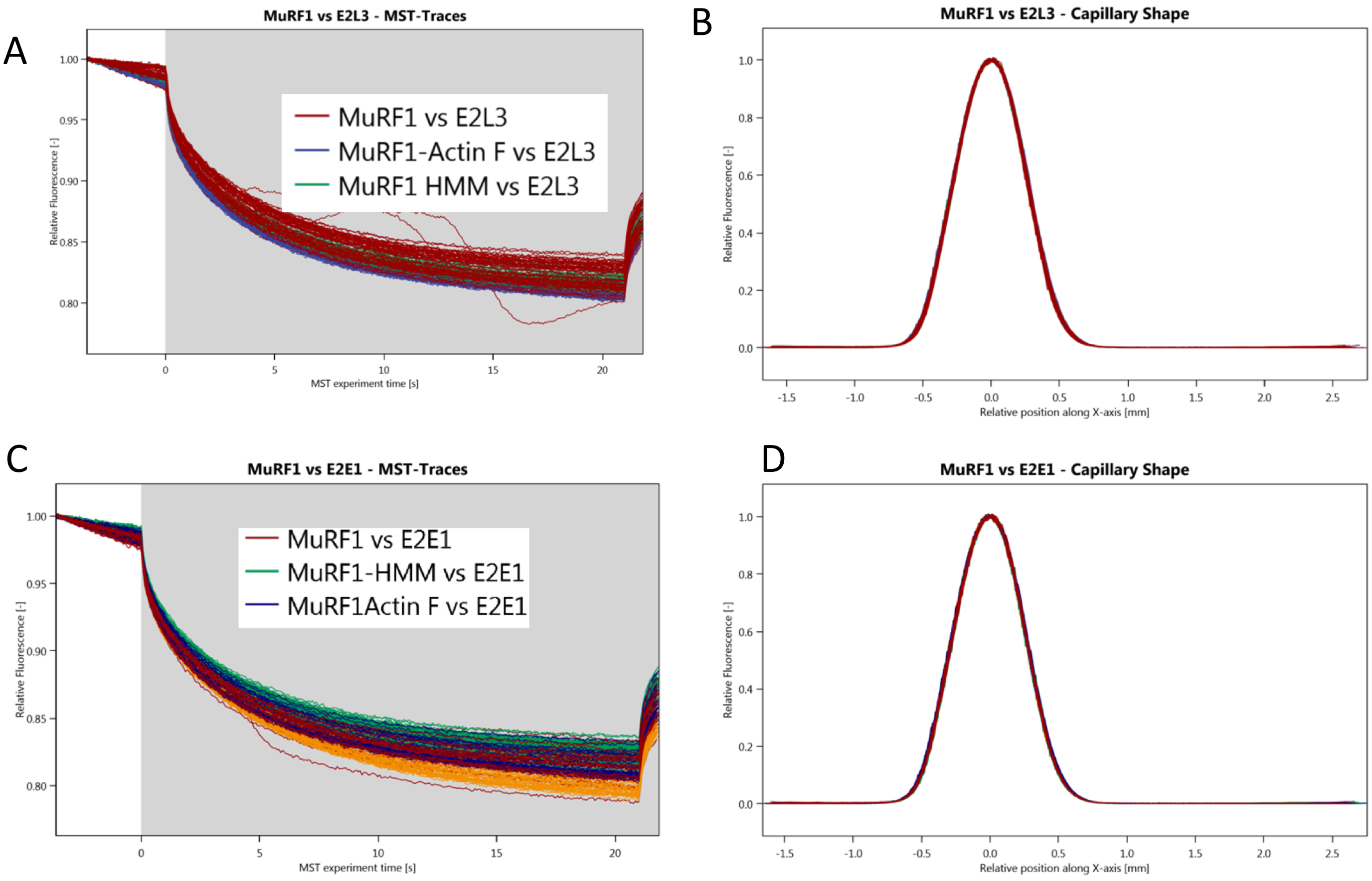

Figure S5. Additional data to Figures 5a and 5c, MST experiments control

A. MST-Trace Chart for MuRF1 +/- substrates vs E2L3 (see legend of Figure 3 for details).
B. Capillary shape for MuRF1 +/- substrates vs E2L3. We verified that fluorescent NT647-MuRF1 was not adsorbed onto the 48 capillaries ((16 for MuRF1 vs E2L3, 16 for MuRF1-Actin vs E2L3 and 16 for MuRF1-HMM vs E2L3) as shown by the presence of the single peak and the perfect overlapping of the curves.
C. MST-Trace Chart for MuRF1 +/- substrates vs E2E1.
D. Capillary shape for MuRF1 +/- substrates vs E2E1. We verified that fluorescent NT647-MuRF1 was not adsorbed onto the 48 capillaries ((16 for MuRF1 vs E2E1, 16 for MuRF1-Actin vs E2E1 and 16 for MuRF1-HMM vs E2E1) as shown by the presence of the single peak and the perfect overlapping of the curves.

Table S1: List of sequences of shRNAs targeting E2L3, cloned into the BLOCK-iT Pol II miR RNAi Expression Vector Kit with EmGFP (Invitrogen number K4936-00).

	**Sequences**
shE2L3-1 Top	TGCTGTTAAAGACATCACTAAGCTGGGTTTTGGCCACTGACTGACCCA GCTTAGATGTCTTTAA
shE2L3-1 Bottom	CCTGTTAAAGACATCTAAGCTGGGTCAGTCAGTGGCCAAAACCCAGC TTAGTGATGTCTTTAAC
shE2L3-2 Top	TGCTGATAACAGGTTCCCTGCAGAGAGTTTTGGCCACTGACTGACTCT CTGCAGAACCTGTTAT
shE2L3-2 Bottom	CCTGATAACAGGTTCTGCAGAGAGTCAGTCAGTGGCCAAAACTCTCT GCAGGGAACCTGTTATC
